# In Vitro Anticancer Activity and Structural Characterization of Ubiquinones from *Antrodia cinnamomea* Mycelium

**DOI:** 10.3390/molecules22050747

**Published:** 2017-05-06

**Authors:** I-Chuan Yen, Shih-Yu Lee, Kuen-Tze Lin, Feng-Yi Lai, Mao-Tien Kuo, Wen-Liang Chang

**Affiliations:** 1 Graduate Institute of Medical Science, National Defense Medical Center, No.116, Sec.6, Minchuan East Road, Neihu District, Taipei 114, Taiwan; yenichuan@mail.ndmctsgh.edu.tw; 2 School of Pharmacy, National Defense Medical Center, Taipei 114, Taiwan; 3 Graduate Institute of Aerospace and Undersea Medicine, National Defense Medical Center, Taipei 114, Taiwan; leeshihyuno1@mail.ndmctsgh.edu.tw (S.-Y.L.); rocodo29@hotmail.com (F.-Y.L.); 4 Department of Radiation Oncology, Tri-Service General Hospital, National Defense Medical Center, Taipei 114, Taiwan; holyholm@mail.ndmctsgh.edu.tw; 5 LanTyng Biotech, Co, Ltd., Taipei 114, Taiwan; maotien.kuo@gmail.com

**Keywords:** *Antrodia cinnamomea*, ubiquinone derivative, antrocinnamone, 4-acetylantrocamol LT3, HPLC analysis and fingerprint, cytotoxicity, flow cytometry, cell cycle

## Abstract

Two new ubiquinones, named antrocinnamone and 4-acetylantrocamol LT3, were isolated along with six known ubiquinones from *Antrodia cinnamomea* (Polyporaceae) mycelium. The developed HPLC analysis methods successfully identified eight different ubiquinones, two benzenoids, and one maleic acid derivative from *A. cinnamomea*. The ubiquinones **1**–**8** exhibited potential and selective cytotoxic activity against three human cancer cell lines, with IC_50_ values ranging from 0.001 to 35.883 μM. We suggest that the different cytotoxicity levels were related to their chemical structures, especially the 4-hydroxycyclohex-2-enone ring and the presence of a free hydroxyl group in the side chain. The suppression by 4-acetylantrocamol LT3 stopped the cell cycle at the beginning of the G2-M phase thus making the cell cycle arrest at the sub-G1 phase as compared with control cells.

## 1. Introduction

Cancer is a significant cause of death worldwide, accounting for some 8.8 million deaths in 2015 [1]. Today numerous new cytotoxic agents are being used as anti-cancer agents, but only small improvements in the survival rate of advanced or metastatic cancer have been observed and new chemotherapeutic drugs are urgently needed. Studies on natural products are one approach for the discovery of new cancer drugs. Only a few ubiquinones with cyclohex-2-enone fragments have been isolated from natural products and the relationships between their cytotoxicity and structures are not clear. These results prompted us to explore further analogs of ubiquinone and compare the effect of different substituents on the cytotoxic activity against some human cancer cell lines.

*Antrodia cinnamomea*, belonging to the Polyporaceae family, is a medicinal fungus found in Taiwan. It exhibits anti-cancer, hepatoprotective and immunomodulatory activities [2,3]. Antroquinonol (**5**) is a ubiquinone derivative found in fermented *A. cinnamomea* mycelium. Recent studies have showed its potent activity against cancer, periodontal diseases, cardiovascular diseases, male infertility, and Parkinsonism [4,5,6,7,8,9]. Antroquinonol showed cytotoxic activity against human breast carcinoma and human liver carcinoma cell lines, as well as against human prostate carcinoma cell lines [10]. In some studies on Chinese herbs introduced in cancer treatment, the crude alcohol extract from *A. cinnamomea* mycelium was found to exhibit major cytotoxic activity on human cancer cell lines [2]. The alcohol extract of this fungi was re-investigated, leading to the characterization and separation of two new ubiquinone derivatives, antrocinnamone (**1**) and 4-acetylantrocamol LT3 (**2**), six known ubiquinone derivatives, two benzenoid derivatives, and one maleic acid derivative. In this study, we report the separation of new ubiquinones from *A. cinnamomea*, the HPLC fingerprint of the alcohol extract of *A. cinnamomea*, and the correlation between respective chemical structure and cytotoxicity of the ubiquinones **1**–**8** on three human cancer cell lines, namely A549, HepG2, and PC3.

## 2. Results and Discussion

### 2.1. Isolation and Identification

**Figure 1:**
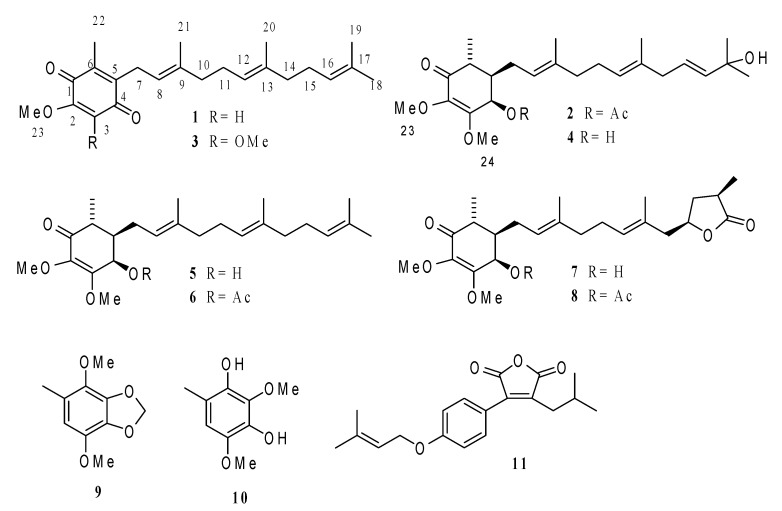
Chemical structures of compounds **1**–**11**.

The dried *A. cinnamomea* mycelium was extracted with 95% alcohol, and the extract was successively partitioned with dichloromethane and water. The dichloromethane-soluble fraction (60 g, dried, from 1305.3 g of mycelium) was subjected to column chromatography and HPLC separation. This procedure afforded eleven compounds **1**–**11** (Figure 1). In all two new ubiquinones, antrocinnamone (**1**) and 4-acetylantrocamol LT3 (**2**), together with nine known compounds, including six known ubiquinones, quinone Q3 (**3**) [11], antrocamol LT3 (**4**) [12], antroquinonol (**5**) [10], 4-acetylantroquinonol (**6**), antroquinonol B (**7**) [13], and 4-acetylantroquinonol B (**8**) [13], two benzenoids, 4,7-dimethoxy-5-methyl-1,3-benzodioxole (**9**) [14] and 2,4-dimethoxy-6-methyl-benzene-1,3-diol (**10**) [14], and one maleic acid derivative, antrodin A (**11**) [15], were identified by comparing their spectral data with literature values.

**Figure 2:**
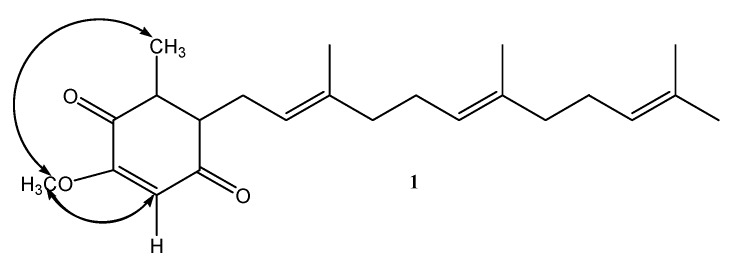
Key NOE correlations for **1**.

Compound **1** was obtained as a yellow oil with molecular formula C_23_H_32_O_3_ from HRESIMS (Supplementary Materials), combined with ^1^H- and ^13^C-NMR spectroscopic data (CDCl_3_, Table 1 and Table 2). Its mass was 30 amu less than that of quinone Q3 (**3**) [11], indicating **1** demethoxylation of quinone Q3. The IR and UV spectra of **1** were similar to those of quinone Q3 (**3**) [11], indicating the presence of a ubiquinone fragment. The ^1^H-NMR spectrum of **1** (CDCl_3_, Table 1) showed five primary methyl groups, one methoxyl group, four methylene groups, and four olefinic protons, which was very similar to **2** except for the absence of an H-24 (δ 3.96, s, 3H) signal in quinone Q3 (**3**) [11]. These results confirmed demethoxylation at C-3, based on the key HMBC correlations observed between H-3 (δ 5.85, s) to C-1 (δ 182.80), C-2 (δ 158.31), and C-5 (δ 138.75) and H-7 (δ 3.19 (2H, d, *J* = 6.8)) to C-4 (δ 186.95), C-5 (δ 138.75), C-6 (δ 144.09), C-8 (δ 118.98), and C-9 (δ 137.51). The stereochemistry of **1** was established from NOESY experiment (Figure 2). The above data suggested **1** to be 5-methoxy-3-methyl-2-(3,7,11-trimethyldodeca-2,6,10-trienyl)cyclohexa-2,5-diene-1,4-dione.

Compound **2** was also obtained as a yellow oil with the molecular formula C_26_H_40_O_6_ from HRESIMS (Supplementary Materials) combined with ^1^H- and ^13^C-NMR spectroscopic data (CDCl_3_, Table 1 and Table 2). The UV spectrum of **2**, which displayed absorption maximum at 264.4 nm, and its IR spectrum (3448, 2974, 1744, 1632, 1454, 1364, 1236, 1142, 1012, 944, 596 cm^−1^) were similar to those of antrocamol LT3 (**4**) [12], indicating the presence of a ubiquinone fragment. The ^1^H-NMR spectrum of **2** (Table 1) showed five primary methyl groups, four methylene groups, two methine protons, two methoxyl groups, one oxygenated methine proton, four olefinic protons, and one acetyl group, which was very similar to **2** except for the presence of an H-4 (δ 5.74, d, 1H) signal further downfield in **4** and an additional acetyl group (δ 2.08, s, 3H) suggesting an additional acetyl group link to C-4 of antrocamol LT3 (**4**) [12].

This was partially established by a COSY-45 spectrum, which indicated the coupling pattern of the oxygenated methine proton (δ 5.74, d, *J* = 3.2 Hz, H-4) and a methine proton at δ 1.88 (1H, m, H-5). The above data suggested **2** to be 4-acetylantrocamol LT3. The configuration of both C-8 and C-12 was the *E* form, as suggested by the δ_C_ of C-20 and C-21 at δ_C_ 16.02 and 16.08, respectively, and in contrast to those of the *Z* configuration at approximately δ_C_ = 25.0 [16]. The relative configuration of the ring in **2** was consistent with that of antroquinonol and was corroborated by a NOESY spectrum [10,12].

### 2.2. HPLC Analysis and Fingerprint of Compounds

The calibration curve of antrocinnamone was linear within a range of 0.125–2.0 mg/mL (R_2_ = 0.9962) and the calibration curve of 4-acetylantrocamol LT3 was linear within a range of 0.125–2.0 mg/mL (R_2_ = 0.9999).

**Figure 3:**
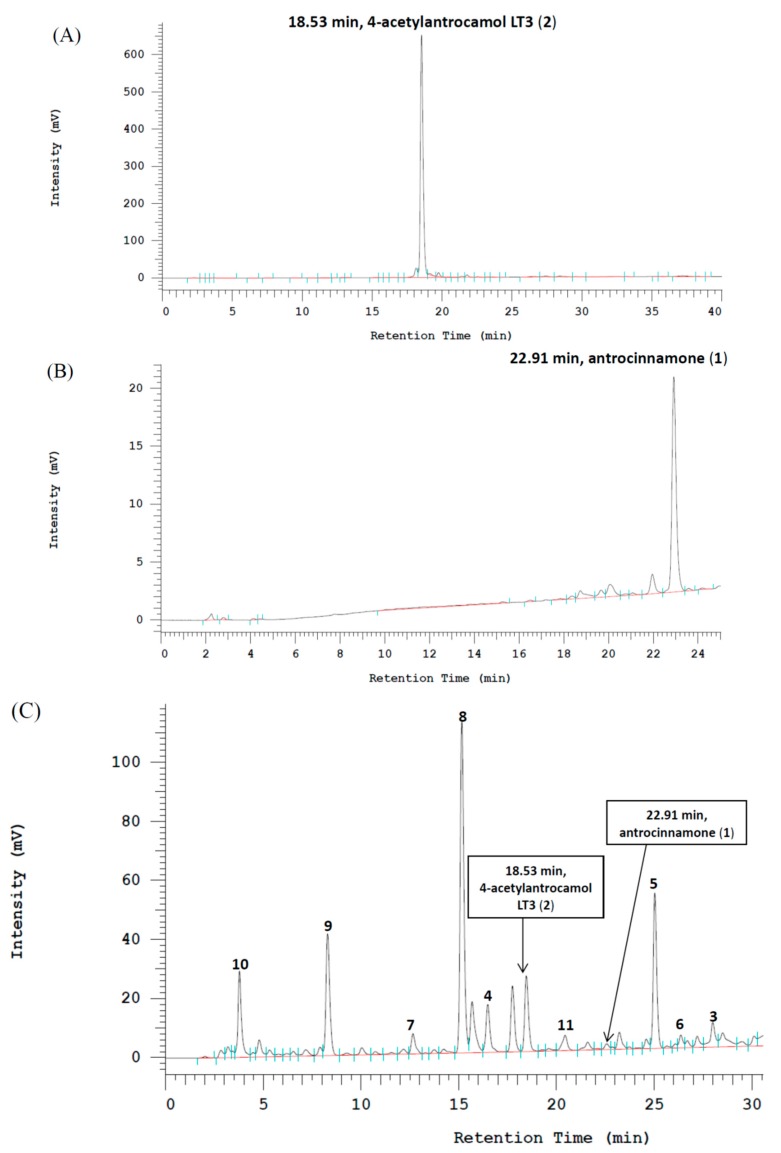
HPLC profiling data of antrocinnamone (**A**), 4-acetylantrocamol LT3 (**B**) and the fingerprint of *Antrodia cinnamomea* ethanolic extract with the retention time of compounds **1**–**11** in HPLC analysis (**C**).

HPLC figures showed that the *A. cinnamomea* ethanolic extract contained 0.64% antrocinnamone and 0.76% 4-acetylantrocamol LT3 (Figure 3A,B). The compounds **1**–**11** were confirmed on the basis of retention time and UV absorption at 254 nm (Figure 3C).

### 2.3. Cytotoxic Activity of Compounds ***1**–**8***

The cytotoxicity evaluation of ubiquinone derivative compounds **1**–**8** against three human cancer cell lines (A549, HepG2, and PC3) showed that the IC_50_ values for these compounds ranged from 0.001 to 100 μM, whereas those against MDCK (a non-malignant cell line) were > 100 μM for most of the compounds (Table 3). Vepesid and taxol were used as positive controls.

In particular antrocamol LT3 (**4**) was the most powerful compound against these three cancer cell lines, with IC_50_ values of 0.080, 0.1060, and 0.0010 μM for the A549, HepG2, and PC3 cell lines, respectively. Ubiquinones possessing a 4-hydroxy-2,3-dimethoxy-6-methylcyclohex-2-enone ring seemed to exhibit selective and powerful cytotoxic activity against the cancer cells, but the acetylation at C-4, e.g., 4-acetylantrocamol LT3 (**2**), reduced the cytotoxicity of ubiquinones. All compounds showed relatively lower toxicity against the non-malignant cell line (MDCK) than the cancer cell lines. Compounds **9**–**11** exhibited no cytotoxicity against normal and cancer cells.

Comparing the ubiquinone derivatives **1**–**8**, we found that different cytotoxicity levels were related to their chemical structure. First, a 4-hydroxycyclohex-2-enone ring may be an essential fragment for the anticancer activity of these ubiquinones. Second, ubiquinones possessing a 4-hydroxycyclohex-2-enone ring and a 4-acetate fragment were much less active relative to the corresponding parent compounds, such as **2**, **6**, and **8**. Third, ubiquinones possessing a 4-hydroxy-cyclohex-2-enone ring with a free hydroxyl group in the side chain have enhanced anticancer activity, such as compound **3** with a 17-hydroxyl group and antrocamol LT1 with an 15-hydroxyl group [2]. Fourth, ubiquinones with a paraquinone may be more selective against PC3 and A549 cells, but not against the HepG2 cell line. Concurrently, compound **3** also has lower toxicity against the normal cell line (MDCK cell line) compared to compound **5**. Compound **7** with a 3-methylfuran-2-one moiety exhibited poorer activity against cancer cell lines than compound **3**. However, compound **7** was less toxic to the normal cell line (MDCK cell line) than compound **5**.

According to the above results, a 4-hydroxycyclohex-2-enone ring contributes to the anticancer activity of these ubiquinone derivatives. Interestingly, the presence of a free hydroxyl group in the side chain significantly increased the anticancer activity compared to antroquinonol and a 4-acetate. Compounds **1**–**8** seemed to exhibit selective cytotoxic activity against the cancer cells. The two benzenoid derivatives **9** and **10**, and the maleic acid **11**, exhibited poor activity against the cancer cell lines, with only compound **9** exhibiting some activity against the PC3 cell line with an IC_50_ value of 13.2590 μM (Table 3). Compounds **1**–**4** and **7**–**11** also showed relatively low cytotoxicity levels against the non-malignant cell line (MDCK) with an IC_50_ > 100 m.

### 2.4. Effect of 4-Acetylantrocamol LT3 on the Cell Cycle Distribution

We initially performed a flow cytometric analysis to confirm whether 4-acetylantrocamol LT3-induced apoptosis was related to arrest the cell cycle progression by quantitating the induction of apoptosis % (sub-G1 phase) and cell cycle distribution.

**Figure 4:**
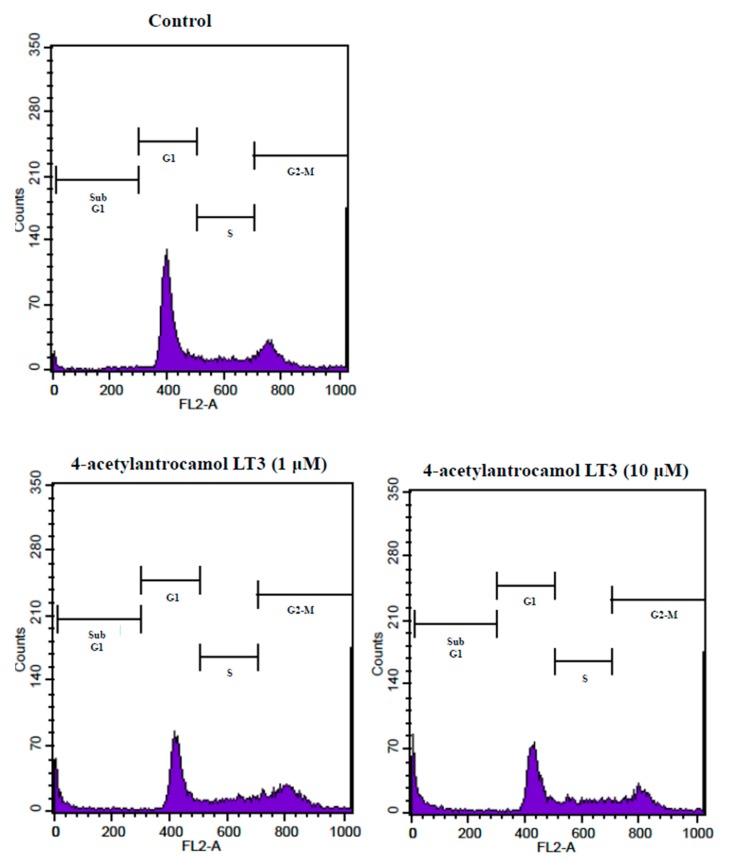
Effect of DMSO (control) and 4-acetylantrocamol LT3 on cell cycle progression of HepG2.

As shown in Table 4 and Figure 4, 4-acetylantrocamol LT3 treatment in HepG2 cells for 24 h induced sub-G1 phase accumulation in a dose dependent manner as compared with control cells. This evidence seems to indicate that 4-acetylantrocamol LT3 could induce HepG2 cell apoptosis.

## 3. Experimental Section

### 3.1. General Methods

Optical rotations were measured on a P-1020 digital polarimeter (JASCO, Tokyo, Japan). UV spectra were recorded on a Multiskan GO spectrophotometer (Thermo Scientific, Vantaa, Finland). IR spectra were obtained using a Nicolet iS5 spectrophotometer (Thermo Scientific, Madison, WI, USA). The 1D and 2D NMR spectra were run on an Agilent spectrometer (400 MHz for ^1^H and 100 MHz for ^13^C) (Agilent Technologies, Santa Clara, CA, USA). Proton and carbon chemical shift values relative to the solvent peak (CDCl_3_, δ_H_ 7.24, δ_C_ 77.0) were used as internal standards. HRESIMS spectra were measured using a Q-TOF AccuTOF GCX GC mass spectrometer (JEOL, Tokyo, Japan). HPLC was performed on a Hitachi instrument equipped with an L-7100 series quaternary gradient pump (Hitachi, Tokyo, Japan), and a diode array detector (L-7455) linked to a Hitachi lachrom software data handling system (D-7000 Multi-HSM-Manager). The system was fitted with a Cosmosil 5C18-MS-II packed HPLC column (A) (250 mm × 4.6 mm I.D. with 5 µm particles) and column (B) 250 mm × 20 mm I.D. with 5 μm particles. The column temperature was maintained at 25 °C and the injection volume was 10 μL. Analytical HPLC was performed using the (A) column under gradient conditions with a mobile phase consisting of a linear gradient from water–methanol (40:60) to water–methanol (20:80) over 15 min, a 15–20 min gradient from water–methanol (20:80) to 100% methanol, and 100% methanol for 10 min at the flow rate of 1.0 mL/min with UV wavelength 254 nm. Preparative HPLC was performed using the (B) column and preparative HPLC conditions were the same as those of analytical HPLC, except that the flow rate was changed from 1 mL/min into 10 mL/min.

### 3.2. Material

Dried mycelium from *A. cinnamomea* fungus was provided by Lan-Tyng Co. (Taipei, Taiwan), in December 2015. The fungi were identified by Prof. H.C. Lin, National Defense Medical Center, Taipei, Taiwan, where a voucher specimen (NDMCP No. 1011201) has been deposited.

### 3.3. Preparation of Crude Fungus Extracts

The dried mycelium of *A. cinnamomea* fungus (6.0 kg) was extracted with 95% ethanol (60 L × 2) at room temperature. The combined extract was concentrated under reduced pressure to yield a brown syrupy mass (**AC-E**; 1305.3 g). This crude extract was dissolved in water and then partitioned (1:1) with dichloromethane to obtain a dichloromethane-soluble fraction (**AC-E-D**; 553.3 g) and a water-soluble fraction (**AC-E-W**; 773.6 g). The dried extracts were stored at 4 °C in the dark until further purification.

### 3.4. Isolation and Purification of Compounds

**Figure 5:**
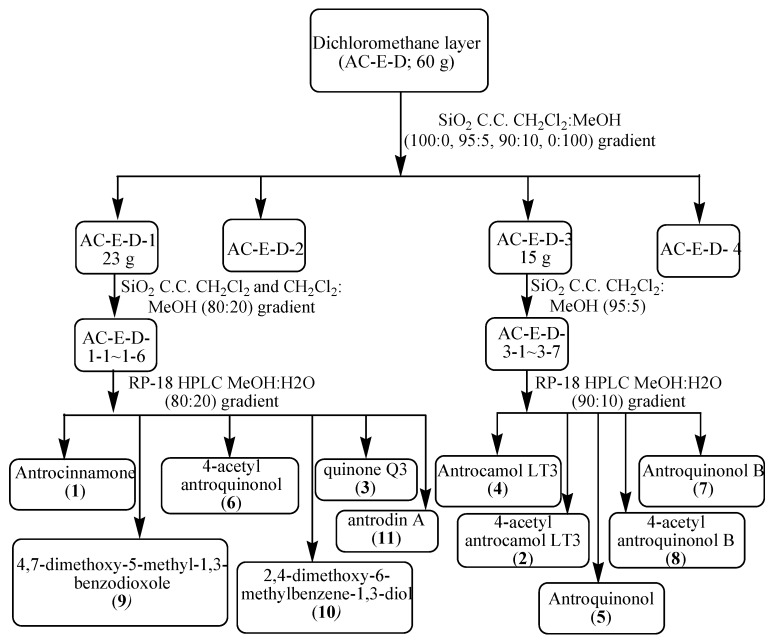
Extraction and isolation of *Antrodia cinnamomea* extract.

The dichloromethane-soluble extract (**AC-E-D**; 60 g) was subjected to column chromatography over silica gel (230–400 mesh) and eluted using a step gradient of dichloromethane-methanol (100:0, 95:5, 90:10, 0:100), which were combined based on TLC profiles to give four major fractions. Column chromatography of the first fraction (**AC-E-D-1**; 23 g) on silica gel (20 × 4 cm) with a linear gradient elution of dichloromethane and dichloromethane-methanol (80:20) yielded six fractions (1–6). The fractions obtained were further purified by preparative HPLC to yield antrocinnamone (**1**; 25.3 mg), quinone Q3 (**3**; 118.4 mg), 4-acetylantroquinonol (**6**; 10.6 mg), 4,7-dimethoxy-5-methyl- 1,3-benzodioxole (**9**; 117.6 mg), 2,4-dimethoxy-6-methylbenzene-1,3-diol (**10**; 9.4 mg), and antrodin A (**11**; 21.3 mg) (Figure 5). The third fraction (**AC-E-D-3**; 15 g) was subjected to silica gel column chromatography (20 × 4 cm) using dichloromethane-methanol (95:5) as eluents to yield seven fractions 1–7. The fractions were further purified by preparative HPLC to yield 4-acetylantrocamol LT3 (**2**; 224.9 mg), antrocamol LT3 (**4**; 153.0 mg), antroquinonol (**5**; 50.9 mg), antroquinonol B (**7**; 65.3 mg), and 4-acetylantroquinonol B (**8**; 10.6 mg) (Figure 5). The chemical structures of these compounds are shown in Figure 1. Antrocinnamone (**1**) and 4-acetylantrocamol LT3 (**2**) are new compounds.

*Antrocinnamone* (**1**): yellow oil; [α${]}_{D}^{23}$ +36.7° (*c* 0.3, MeOH); UV (MeOH) λ_max_ (log ε): 267.2 (2.0) nm; ESIMS (positive) *m*/*z* 357.2 [M + H]^+^; IR (KBr) ν_max_: 3455, 2065, 1644, 1441, 1224, 554 cm^−1^; ^1^H- and ^13^C-NMR, see Table 1 and Table 2; HRESIMS *m*/*z* 356.2342 (calcd. for C_23_H_32_O_3_ [M]^+^ 356.2346).

*4-Acetylantrocamol Lt3* (**2**): yellow oil; [α${]}_{D}^{23}$ +114.7° (*c* 0.3, MeOH); UV (MeOH) λ_max_ (log ε): 264.4 (0.7) nm; HRESIMS *m*/*z* 448.2827 (calcd. for C_26_H_40_O_6_ [M]^+^ 448.2819); IR (KBr) ν_max_: 3448, 2974, 1744, 1632, 1454, 1364, 1236, 1142, 1012, 944, 596 cm^−1^; ^1^H- and ^13^C-NMR, see Table 1 and Table 2.

### 3.5. Preparation of Standard and Sample Solutions for HPLC Analysis

Standard stock solutions were prepared by dissolving ethanolic extract and compounds **1**–**11** in methanol and storing them at 4 °C in a refrigerator. The reference standards were prepared by serial dilutions prior to use. Finally, the *A. cinnamomea* ethanolic extract dissolved in methanol (10 mg/mL) and compounds **1**–**11** dissolved in methanol (a range of 0.125–2.0 mg/mL) were prepared for HPLC analysis. These solutions was filtered through a disposable syringe filter (0.50 mm, Dismic-25JP Advantec, Yamanashi, Japan) before injection into an HPLC system.

### 3.6. HPLC Analysis Method

Constituents of *A. cinnamomea* ethanolic extract were analysed by HPLC. The eluted components were identified based on the retention time in comparison with the used reference standards. The identification of constituents was also confirmed with a photodiode array detector by comparison with UV spectra of standards over the wavelength range 254 nm.

### 3.7. Chemicals and Reagents

Dimethyl sulfoxide (DMSO), ethylene diaminetetraacetic acid (EDTA), fetal bovine serum (FBS), l-glutamine penicillin–streptomycin solution (GPSS), Hank’s buffered saline solution (HBSS), potassium phosphate monobasic (KH_2_PO_4_), potassium phosphate dibasic (K_2_HPO·3H_2_O), Roswell Park Memorial Institute-1640 medium (RPMI-1640), trypsin-EDTA, and Tris base were obtained from Sigma (St. Louis, MO, USA). Cell Counting Kit-8 (CCK-8) were purchased from Dojindo Molecular Technologies, Inc. (Kumamoto, Japan).

### 3.8. Cell Lines and Culture

Three human cancer cell lines (A549, HepG2 and PC3) and normal cell line (MDCK) were obtained from the ATCC (Manassas, VA, USA) and grown on Eagle’s minimum essential medium (EMEM, GIBCO, Waltham, MA, USA) supplemented with F12 medium containing 10% FBS, 100 IU/mL penicillin and 100 μg/mL streptomycin [17].

### 3.9. Cytotoxicity Analysis Method

Viability of the human cancer cell lines and normal cell line treated with different concentrations of *A. cinnamomea* extracts, subfractions, and isolates was evaluated using the cell counting kit-8 assay. Briefly, a total of 5 × 10^3^ cells/well (A549, HepG2, PC3, or MDCK cells) were seeded into 96-well plates for 12 h, and the cells were treated with various concentrations of the isolated compounds (0.01–100 μM, double dilution method) for 24 h. The original medium was removed, 10% of the cell counting kit-8 in PBS was added to each well, and the plates were incubated for 2 h. The absorbance was measured at 540 nm using an enzyme-linked immunosorbent assay (ELISA) plate reader [18].

### 3.10. Cell Cycle Analysis

Stock solutions of 4-acetylantrocamol LT3 (20 mM) were prepared by dissolving them in DMSO, and appropriate working dilutions were prepared with the cell culture medium prior to the experiments. For cell cycle distribution analysis, HepG2 cells were treated with DMSO (control) and 4-acetylantrocamol LT3 for 24 h. The cells were then fixed in ice-cold 70% methanol for overnight. After an additional wash with PBS, the cell pellets were stained with 1 mL of propidium iodide (PI) staining solution containing 200 μg of PI in 1 mL of PBS containing 2 mg of DNase free RNase for 30 min. Acquisition and analysis were performed by flow cytometry (FACSCalibur flow cytometer, Becton, Dickinson and Co., Franklin Lakes, NJ, USA) with excitation at 488 nm. Data on each sample represent the percentage of cells in the sub-G1, G1, S, G2/M phases of the cell cycle, respectively.

### 3.11. Statistical Analysis

All experiments were carried out in triplicate, and each experiment was repeated three times. Data analysis was carried out using SPSS statistical package version 18.0 (SPSS, Inc., Armonk, NY, USA). The IC_50_ values (the concentration of drug necessary to induce 50% inhibition) were measured for all of the tested drugs using the Probit test in the SPSS software. The cell cycle distribution values of mean and standard deviation (mean ± SD) and 95% confidence intervals (CI) of means to verify the statistical significance of all parameters were calculated. The *p*-values of < 0.05 were adopted as statistically significant. All data are presented in this report as means ± SD of three replicates from three separate experiments.

## 4. Conclusions

In conclusion, the present study reports the identification of two new ubiquinones, antrocinnamone (**1**) and 4-acetylantrocamol LT3 (**2**) isolated from *Antrodia cinnamomea* (Polyporaceae) mycelium. In addition, we developed an HPLC analysis method to separate eight different ubiquinones from *A. cinnamomea* and compared their cytotoxic activity against human cancer cell lines. We found that the different cytotoxicity levels were related to their chemical structures, where especially a 4-hydroxycyclohex-2-enone ring and the presence of a free hydroxyl group in the side chain, could potentiate the anticancer activity on three human cancer cell lines, A549, HepG2, and PC3. 4-Acetylantrocamol LT3 treatment in HepG2 cells for 24 h induced about 4–7-fold sub-G1 phase accumulation in a dose dependent manner as compared with control cells. We suggest that suppression by 4-acetylantrocamol LT3 blocked the entry of the cell cycle at the G2-M phase and thus caused cell cycle arrest at the sub-G1 phase as compared with control cells.

## Figures and Tables

**Table 1:** ^1^H-NMR spectroscopic data (CDCl_3_) for compounds **1**–**4**.

Position	1	3	4	2 ^a^
	δ_H_ Mult. (*J* in Hz)	δ_H_ Mult. (*J* in Hz)	δ_H_ Mult. (*J* in Hz)	δ_H_ Mult. (*J* in Hz)
3	5.85 s			
4			4.31 d (2.8)	5.71 d (3.2)
5			1.70 m	1.88 m
6			2.51 dq (9.6, 6.8)	2.51 dq (10.8, 7.2)
7	3.19 d (6.8)	3.16 d (6.8)	2.21 m	2.24 m 1.95 m
8	4.92 t (6.8)	4.91 t (6.8)	5.14 t (6.8)	5.09 t (6.8)
10	1.90–2.00 m	1.90–2.00 m	2.02 m	2.00 m
11	1.97–2.05 m	1.97–2.05 m	2.08 t	2.06 m
12	5.04 t (7.2)	5.03 t (7.2)	5.08 t (6.4)	5.09 t (6.8)
14	1.90–2.00 m	1.90–2.00 m	2.64 d (4.8)	2.63 d (5.6)
15	1.97–2.05 m	1.97–2.05 m	5.56 m	5.57 m
16	5.05 t (7.2)	5.05 t (7.2)	5.56 m	5.59 d (5.6)
18	1.66 s	1.65 s	1.29 s	1.29 s
19	1.56 s	1.57 s	1.29 s	1.29 s
20	1.55 s	1.55 s	1.55 s	1.53 s
21	1.72 s	1.72 s	1.63 s	1.55 s
22	2.02 s	2.00 s	1.14 d (7.2)	1.17 d (7.2)
23	3.77 s	3.97 s	3.63 s	3.65 s
24		3.96 s	4.04 s	3.98 s

^a^ Acetate group: δ 2.08 (s, OC=OCH_3_).

**Table 2:** ^13^C-NMR spectroscopic data (CDCl_3_) for compounds **1**–**4**.

Position	1	3	4	2 ^a^
	δ_C_ Mult.	δ_C_ Mult.	δ_C_ Mult.	δ_C_ Mult.
1	182.80 s	184.75 s	197.20 s	196.91 s
2	158.31 s	144.36 s	135.84 s	137.29 s
3	107.09 s	144.21 s	160.58 s	158.20 s
4	186.95 s	183.90 s	67.88 d	69.10 d
5	138.75 s	138.85 s	43.40 d	43.00 d
6	144.09 s	141.67 s	40.24 d	41.29 d
7	25.68 t	25.29 t	26.94 t	26.84 t
8	118.98 d	118.87 d	121.19 d	120.36 d
9	137.51 s	137.57 s	137.76 s	137.77 s
10	39.68 t	39.68 t	39.62 t	39.60 t
11	26.43 t	26.45 t	26.35 t	26.44 t
12	123.82 d	123.83 d	124.83 d	124.90 d
13	135.19 s	135.18 s	134.04 s	133.80 s
14	39.66 t	39.67 t	42.20 t	42.29 t
15	26.74 d	26.72 d	125.22 d	125.26 d
16	124.30 d	124.29 d	139.16 d	139.20 d
17	131.29 s	131.27 s	70.76 s	70.63 s
18	25.48 q	25.67 q	29.85 q	29.77 q
19	17.65 q	17.64 q	29.80 q	29.77 q
20	16.00 q	15.98 q	16.14 q	16.02 q
21	16.34 q	16.30 q	16.14 q	16.08 q
22	11.85 q	11.92 s	12.32 q	12.83 q
23	56.07 q	61.12 q	60.59 q	60.69 q
24		61.12 q	59.20 q	59.67 q

^a^ Acetate group: δ 169.77 (s, C=O), 20.94 (q, CH_3_).

**Table 3:** Cytotoxic activity of compounds **1**–**11** derived from *Antrodia cinnamomea*.

Compound	IC50 (μM)			
	MDCK ^a^	A549 ^a^	HepG2 ^a^	PC3 ^a^
**1**	>100	0.3820	>100	0.0140
**2**	>100	0.0270	0.1280	4.2850
**3**	>100	4.1600	>100	0.0600
**4**	>100	0.0080	0.1060	0.0010
**5**	10.531	0.4210	0.0440	0.0730
**6**	41.612	0.6110	0.0750	2.3150
**7**	>100	6.0320	21.3720	1.0310
**8**	>100	35.8830	>100	20.3310
**9**	>100	>100	>100	13.2590
**10**	>100	>100	>100	>100
**11**	>100	>100	90.4110	>100
**Vepesid^®^ (μ** **M** **)**			4.049	
**Taxol^®^ (n** **M** **)**		0.354		0.461

^a^ The results shown here represent the means (*n* = 3).

**Table 4:** Changes of cell cycle progression by 4-acetylantrocamol LT3 in HepG2 cancer cells.

Dosage (μM)	sub G1 (%)	G1 (%)	S (%)	G2-M (%)
Control ^a,b,c^	1.2 ± 0.4	53.2 ± 1.8	13.2 ± 3.4	31.3 ± 2.5
4-acetylantrocamol LT3 (1 μM) ^a,b,c^	6.0 ± 0.7	37.4 ± 4.2	15.4 ± 1.6	33.6 ± 4.7
4-acetylantrocamol LT3 (10 μM) ^a,b,c^	9.2 ± 2.1	32.9 ± 2.4	19.3 ± 4.9	33.6 ± 5.6

^a^ HepG2 cells were treated with increasing concentrations of 4-acetylantrocamol LT3 and DMSO (control) for 24 h. ^b^ The distribution of cells at each phase of cell cycle was analyzed by flow cytometry. ^c^ All data presented in this table are means ± SD of three replicates from three separate experiments.

## Supplementary Materials

Supplementary materials are available online.
