# Supplementary material for: In Vitro Anticancer Activity and Structural Characterization of Ubiquinones from Antrodia cinnamomea Mycelium
Source: Molecules. 2017 May 6;22(5):747. doi: 10.3390/molecules22050747 (PMC6154633; doi:10.3390/molecules22050747)
Supplement: Supplementary file 1 [file molecules-22-00747-s001.pdf]

In Vitro Anticancer Activity and Structural Characterization of Ubiquinones from *Antrodia cinnamomea* Mycelium

I-Chuan Yen [1,5], Shih-Yu Lee [2], Kuen-Tze Lin [3], Feng-Yi Lai [2], Mao-Tien Kuo [4], Wen-Liang Chang [5,*]

Compound **1:** antrocinnamone

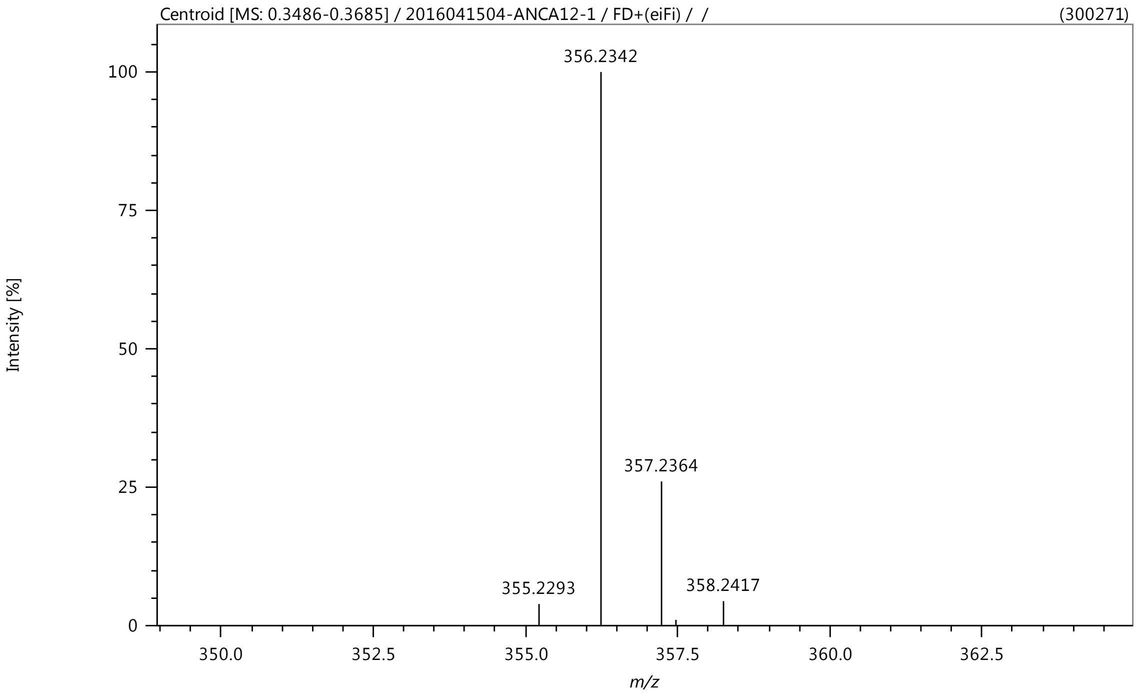

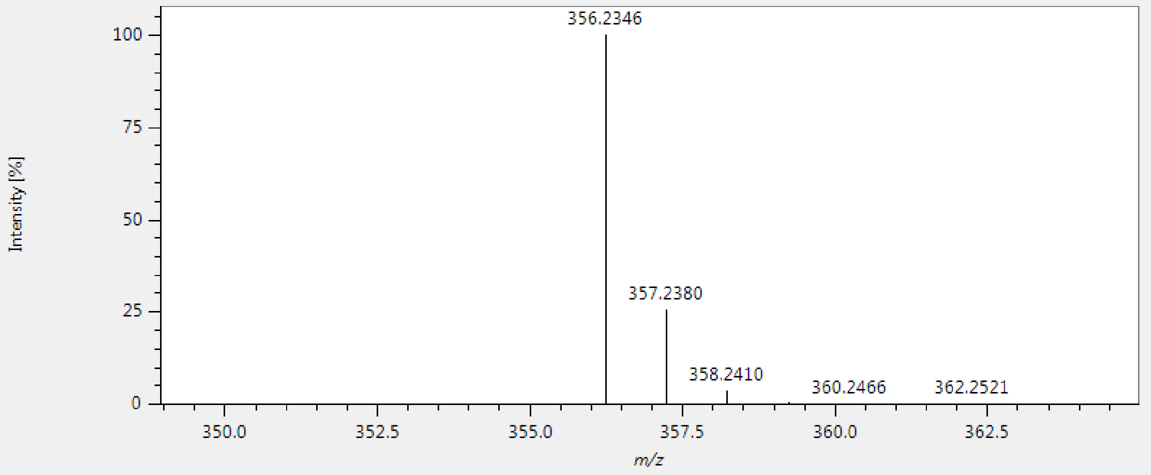

Mass	Intensity	Formula	Calculated Mass	Mass Difference [mDa]	Mass Difference [ppm]	DBE
356.23415	300271.46	C23 H32 O3	356.23460	-0.44	-1.24	8.0

Compound **2:** 4-acetylantrocamol LT3

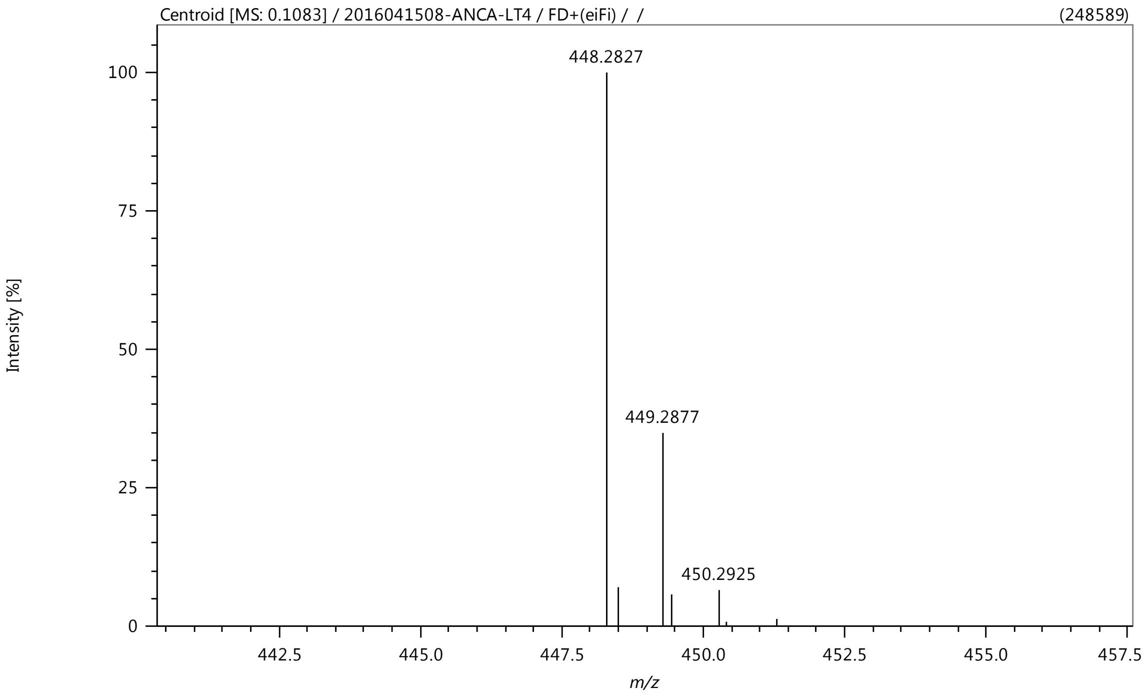

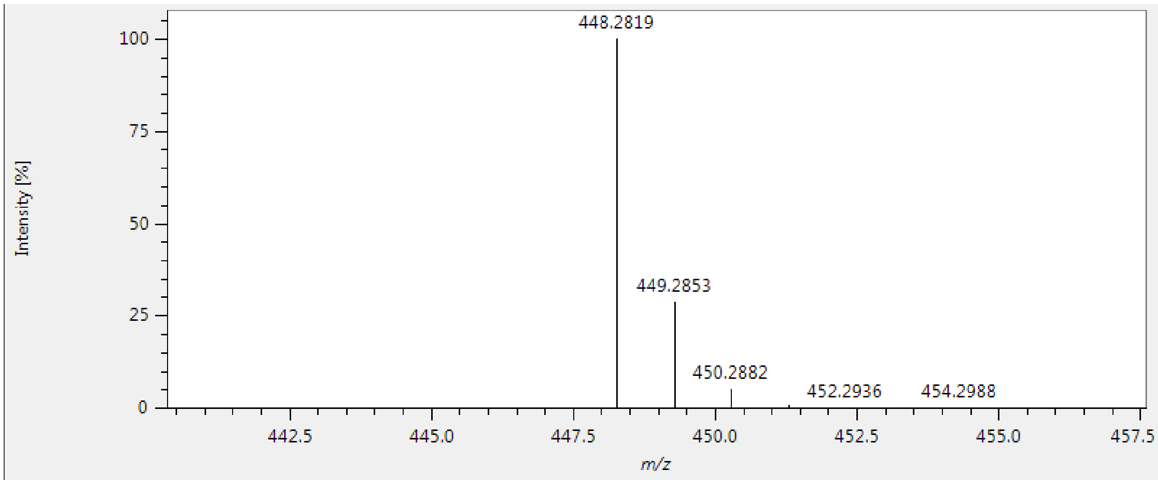

	Mass	Intensity	Formula	Calculated Mass	Mass Difference [mDa]	Mass Difference [ppm]	DBE
▸	448.28273	248589.00	C26 H40 O6	448.28194	0.79	1.77	7.0
